# Knockout of *OsNramp5* using the CRISPR/Cas9 system produces low Cd-accumulating *indica* rice without compromising yield

**DOI:** 10.1038/s41598-017-14832-9

**Published:** 2017-10-31

**Authors:** Li Tang, Bigang Mao, Yaokui Li, Qiming Lv, LiPing Zhang, Caiyan Chen, Hanjie He, Weiping Wang, Xiongfeng Zeng, Ye Shao, Yinlin Pan, Yuanyi Hu, Yan Peng, Xiqin Fu, Hongqing Li, Shitou Xia, Bingran Zhao

**Affiliations:** 1 Hunan Provincial Key Laboratory of Phytohormones and Growth Development, College of Bioscience and Biotechnology, Hunan Agricultural University, Changsha, 410128 China; 2 State Key Laboratory of Hybrid Rice, Hunan Hybrid Rice Research Center, Changsha, 410125 China; 3 Key Laboratory of Agro-Ecological Processes in Subtropical Region, Institute of Subtropical Agriculture, Chinese Academy of Sciences, Changsha, 410125 China; 4 Hunan Provincial Key Laboratory of Forestry Biotechnology, Central South University of Forestry and Technology, Changsha, 410004 China; 5 Guangdong Provincial Key Lab of Biotechnology for Plant Development, South China Normal University, Guangzhou, 510631 China

## Abstract

Rice grain with excessive cadmium (Cd) is a major source of dietary Cd intake and a serious threat to health for people who consume rice as a staple food. The development of elite rice cultivars with consistently low Cd content is challenging for conventional breeding approaches, and new strategies urgently need to be developed. Here, we report the development of new *indica* rice lines with low Cd accumulation and no transgenes by knocking out the metal transporter gene *OsNramp5* using CRISPR/Cas9 system. Hydroponic culture showed that Cd concentrations in shoots and roots of *osnramp5* mutants were dramatically decreased, resulting in rescue of impaired growth in high Cd condition. Cd-contaminated paddy field trials demonstrated that Cd concentration in *osnramp5* grains was consistently less than 0.05 mg/kg, in contrast to high Cd concentrations from 0.33 mg/kg to 2.90 mg/kg in grains of Huazhan (the wild-type *indica* rice). In particular, the plant yield was not significantly affected in *osnramp5* mutants. Furthermore, we developed promising hybrid rice lines with extremely low Cd content in grains. Our work supplies a practical approach to developing Cd pollution-safe *indica* rice cultivars that minimizes Cd contamination risk in grains.

## Introduction

Cadmium (Cd) is a highly toxic heavy metal for most living organisms^1^. The biological half-life of Cd in the human body is estimated to be nearly 30 years^2^. Persistent intake of Cd can lead to chronic kidney toxicity, ‘itai-itai disease’, cancer and other health problems^3^. Rice is an important staple food for more than half of the world’s population^4^, and about 90% of the world’s rice is produced in Asia^5^. A recent survey showed that the mean concentration of Cd in rice grain samples from Asia was higher than in samples from Europe, the Middle East, and North America^6^. In South and Southeast Asia, *indica* rice is the main type produced and consumed^2^. Cd contamination of rice grains in these areas has been reported from China^5^, India^7,8^, Thailand^9^, Indonesia^6,10^, Bangladesh^8^, and Sri Lanka^8^. In general, Cd content in shoots and grains was higher in standard *indica* rice cultivars than in *japonica* rice cultivars^11–14^. Therefore, controlling Cd accumulation in *indica* rice grains is important for food safety and the health of people who consume rice as a staple of their diet.

Rice grains acquire Cd from contaminated paddy fields. Treatments such as soil removal and replacement, chemical washing, or phytoremediation can repair contaminated soil, but all of these methods are expensive and/or time-consuming^15,16^. Some measures such as lime and charcoal application, or field flooding for 3 weeks before and after rice heading, can limit the mobility and bioavailability of Cd. However, these methods may also reduce grain yield and are not long-term solutions, because Cd can be remobilized with changes in soil conditions^17,18^. Selection and breeding of low-Cd accumulation rice cultivars is an economical strategy, and would allow rice production in Cd-polluted soils^5,11,14^.

Understanding the physiological processes and molecular mechanisms of Cd uptake and transport within rice is essential for low-Cd rice breeding. Cd is uptaken by roots from the soil, transported into shoots through xylem flow, and accumulated in leaves and stems. During the reproductive growth phase, most Cd transported by xylem is transferred to phloem in the nodes and is then preferentially transported into upper nodes and grains rather than into leaf blades. These results are based on visualization of Cd in real time by a positron-emitting tracer imaging system (PETIS)^19^. Furthermore, Cd stored in leaf blades is remobilized through phloem and transported into grains after redirection in nodes during the ripening stage^2,15^. Many Cd transporters have been identified and characterized, including: OsIRT1, OsIRT2, and two members of the natural resistance associated macrophage proteins (NRAMP) family, OsNramp5 and OsNramp1, which mediate the root uptake of Cd^20–23^; OsHMA3, which sequesters Cd into vacuoles of root cells^24,25^; OsHMA2, which facilitates xylem loading of Cd^26,27^; and OsLCT1, which plays a role in transferring Cd from xylem to phloem in nodes and transporting Cd by phloem^28^. Modifying these transporter genes may reduce Cd content in rice. Among them, mutating *OsNramp*5 causes the largest reduction in Cd content. In a previous study, *osnramp5* mutants with a background of the *japonica* cultivar Koshihikari were isolated from an ion-beam irradiated population. When grown in Cd-contaminated paddy fields, these mutants accumulated only a slight amount of Cd in grains, ranging from 0.01–0.03 mg/kg, less than 3% of the amount in grains of wild type plants. Notably, *osnramp5* mutants did not show any significant defects of important agronomical traits such as growth, yield, or taste, although the mutants showed a remarkable reduction of manganese (Mn) content^21^. The T-DNA insertion mutant of *OsNramp*5 (with a background of *japonica* cultivar Zhonghua 11) also showed huge reductions in Cd and Mn accumulation, but the grain yield of this mutant was only 11% of the wild-type plants^20^. *Indica* rice tends to accumulate more Cd than *japonica* cultivars, but the effects of *OsNramp*5 mutation on accumulation of Cd and other relevant metals, and on major agronomic traits have not yet been clarified in *indica* rice.

Hybrid rice has a 10–20% yield advantage over conventional rice and has been developed in more than 40 countries worldwide^29,30^. *Indica* hybrids dominate hybrid rice production, especially in southern China. However, a considerable proportion of rice grains contaminated with excess Cd have been found in southern China^5,31^. Thus, genetic improvements in hybrid rice directed at low-Cd accumulation in grains have become a top priority for hybrid rice breeders. So far, there is no Cd pollution-safe rice germplasm in which grains consistently accumulate lower Cd than the national food safety standard of 0.2 mg/kg when grown in high Cd-contaminated paddy fields in China. In 2016, researchers grew 617 *indica* hybrid varieties from the middle and lower Yangtze River Valley in China and 68 inbred rice cultivars from around the world in paddy fields with soil Cd concentration between 1.80 and 2.80 mg/kg. They found that grain Cd levels ranged from 0.67 to 7.83 mg/kg, much higher than the national standard^32^. The deficiency of Cd pollution-safe germplasm has limited the breeding of low-Cd rice cultivars and hybrid rice combinations. Thus, new technologies to minimize the Cd content in rice grains are urgently required to reduce the risk of Cd contamination.

In recent years, genome editing technologies have been successfully used to genetically modify plants^33–38^. Among them, CRISPR/Cas9 (Clustered Regularly Interspaced Short Palindromic Repeats/CRISPR-associated Cas9) has revolutionized genome editing and is widely used because of its simplicity, efficiency, and versatility. However, there are only a few reports to date in which the CRISPR/Cas9 system has been successfully used for rice genetic improvement^30,39–44^.

In this report, we obtained *osnramp5* mutants with backgrounds of Huazhan (HZ) and Longke638S (638S), both of which are extensively used *indica* parental lines in the current two-line hybrid rice breeding. The transgene-free homozygous mutants were efficiently isolated in the T_1_ generation. Notably, the mutants had extremely low-Cd content in grains and did not show stunted growth or reduced yield when grown in highly Cd-contaminated paddy fields. Furthermore, the Cd concentration in grains of Long-liang-you-hua-zhan (LLYHZ or 638 S/HZ), a widely cultivated super-hybrid rice in China, was rapidly and considerably reduced by crossing the HZ mutant and 638S mutant.

## Results

### Site-specific mutagenesis of *OsNramp5* induced by CRISPR/Cas9 system

**Figure 1:**
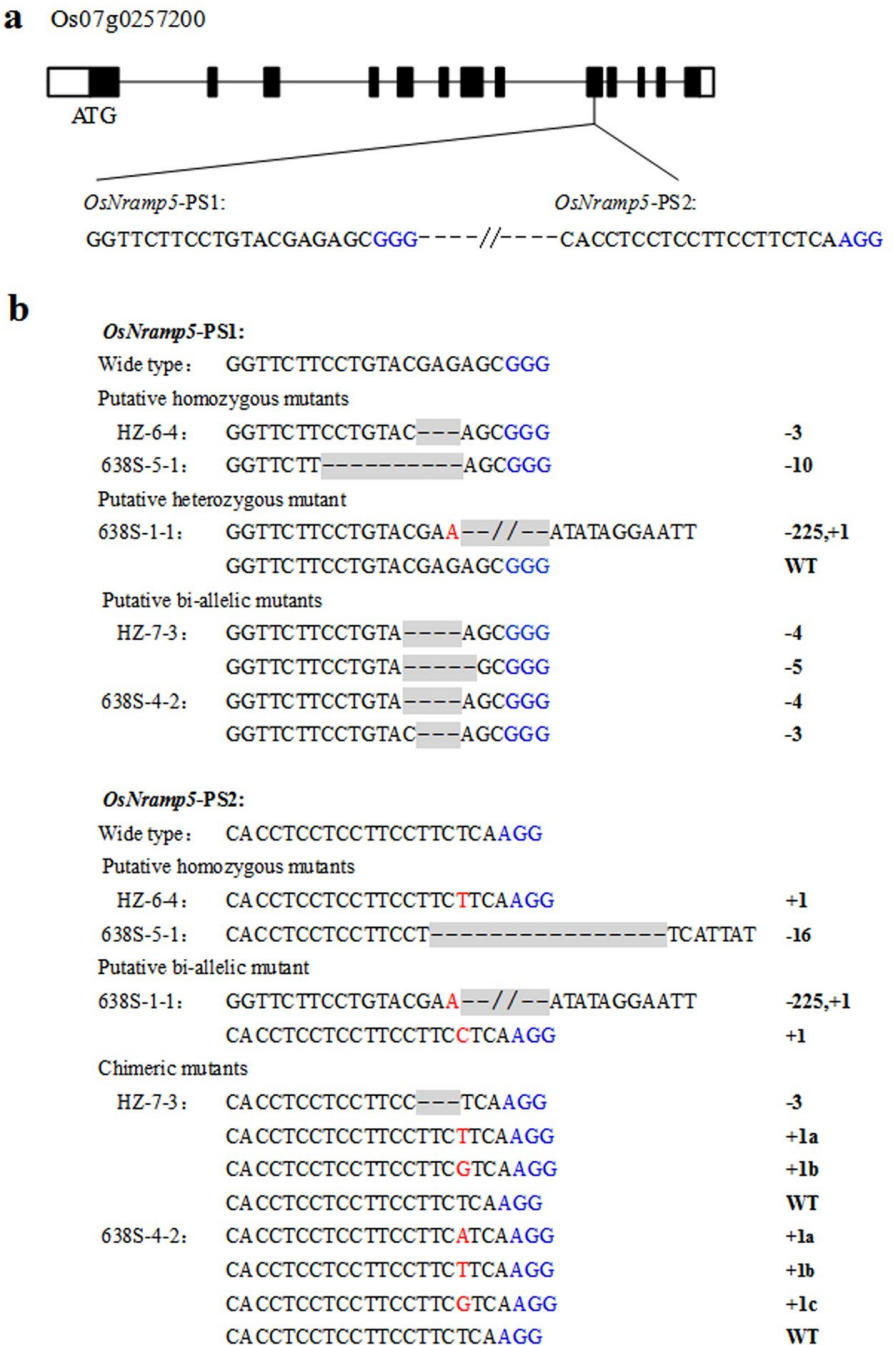
CRISPR/Cas9-induced mutations in the *OsNramp5* gene. **(a)** Schematic diagram of *OsNramp5* gene structure and two CRISPR/Cas9 target sites. UTRs, exons, and introns are indicated by blank rectangles, black rectangles, and black lines, respectively. The 20-nt target sequences are shown at the bottom of the gene structure, and the following PAMs (NGG) are highlighted in blue. **(b)** The mutant *OsNramp5* genotypes of representative T_0_ plants are identified by DNA sequencing and alignment. Deletions and insertions are indicated by dashes and red letters, respectively. The numbers on the right side show the sizes of the indels, with “−” and “+” indicating deletion and insertion of the nucleotides involved, respectively. The letters after the numbers represent different bases of the same length.

We chose to target exon IX of *OsNramp5* for CRISPR/Cas9-based mutagenesis, as mutations in this exon have been shown to result in low Cd concentration of grains in the *japonica* Koshihikari background^21^. We designed two sequence-specific single guide RNA (sgRNA) target sites, *OsNramp5*-PS1 and *OsNramp5*-PS2, which are 119 bp apart in the *OsNramp5* sequence (Fig. 1a). Then they were ligated into two sgRNA expression cassettes of a Cas9 binary vector^45^, driven by OsU6 and OsU3 promoters, respectively. The vector was introduced into HZ and 638S by *Agrobacterium*-mediated transformation. Using site-specific PCR and Sanger sequencing, a total of 14 HZ mutants were recovered from 17 T_0_ transgenic HZ plants (82.4%) and 8 638S mutants were recovered from 10 T_0_ transgenic 638S plants (80.0%). Mutation efficiency of individual target sites was varied and depended on different target sites and backgrounds, ranging from 70.0% to 82.4% (Table 1). The majority of mutations were short insertions and deletions (indels), except for one deletion of a 225 bp DNA fragment spanning the two target sites. Every 4th base upstream of the protospacer adjacent motif (PAM) was mutated, and aligned perfectly with the expected Cas9 cleavage site, which is 3 base pairs upstream of the PAM sequence (Fig. 1b).

**Table 1:** The mutation rates of T_0_ transgenic plants.

Target site	Host cultivar	No. of plants examined	No. of plants with mutations	Mutation rate (%)	Putative homozygous mutations		Putative bi-allelic mutations		Putative heterozygous mutations	
					Number	%	Number	%	Number	%
*OsNramp5*-PS1	Huazhan	17	14	82.4	3	17.6	9	53.0	1	5.9
	Longke 638S	10	8	80.0	1	10.0	6	60.0	1	10.0
*OsNramp5*-PS2	Huazhan	17	12	70.6	3	17.6	5	29.4	1	5.9
	Longke 638S	10	7	70.0	1	10.0	5	50.0	0	0.0

The genotypes of the T_0_ mutants were analysed in detail by sequencing the PCR products of DNA extracted from at least 5 leaves from different tillers during grain maturation. The genotypes of the mutants could be classified into 4 types: 1) homozygotes with both alleles containing the same mutations, including lines HZ-6–4 and 638S-5-1; 2) heterozygotes with only one allele mutated, such as line 638S-1-1 at target site *OsNramp5*-PS1; 3) bi-allelic mutants with both copies of the target sequence mutated differently, including lines HZ-7-3 and 638S-4-2 at target site *OsNramp5*-PS1; and 4) chimeras in which at least 3 distinct target sequences were detected, including lines HZ-7-3 and 638S-4-2 at target site *OsNramp5*-PS2 (Fig. 1b). Overall, bi-allelic mutations occurred with the highest frequency in the T_0_ generation (Table 1).

Off-target mutations in the CRISPR/Cas9 system have been reported in previous studies^46,47^. To determine whether off-target events were produced in our experiments, we examined 4 putative off-target sites in the rice genome that showed the highest sequence similarity with our target sites. Among all 27 T_0_ transgenic plants and 30 T_1_ transgenic plants, no off-target events were found (Supplementary Table 1). These results demonstrate that the sgRNA we designed are of high specificity, and thus we have obtained a high gene editing efficiency with undetectable off-target mutations.

### Obtaining homozygous knockout lines free of transgenes in the T_1_ generation

To further understand the inheritance of mutation, 4 T_0_ plants and their progeny were investigated. For each T_0_ plant, 20–38 progeny were sampled randomly and genotyped individually at the two target sites (Supplementary Table 2). As expected, all of the T_0_ putative homozygotes and their offspring had identical genotypes (HZ-6-4 and 638S-5-1), suggesting that the mutations in these T_0_ plants were faithfully inherited in the next generation. For the T_1_ generation of putative bi-allelic mutants (HZ-7-3 and 638S-4-2 at *OsNramp5*-PS1), the genotype segregation ratio was consistent with Mendelian law, indicating that the targeted mutations in T_0_ plants were inherited normally. For the T_1_ generation of chimeras (HZ-7-3 and 638S-4-2 at *OsNramp5*-PS2), some parental mutations were lost, probably because these mutations took place in parental somatic cells that did not participate in gamete production, and some new mutations were generated, probably due to continuous modification of the wild-type (WT) alleles in Cas9 positive T_1_ lines.

**Figure 2:**
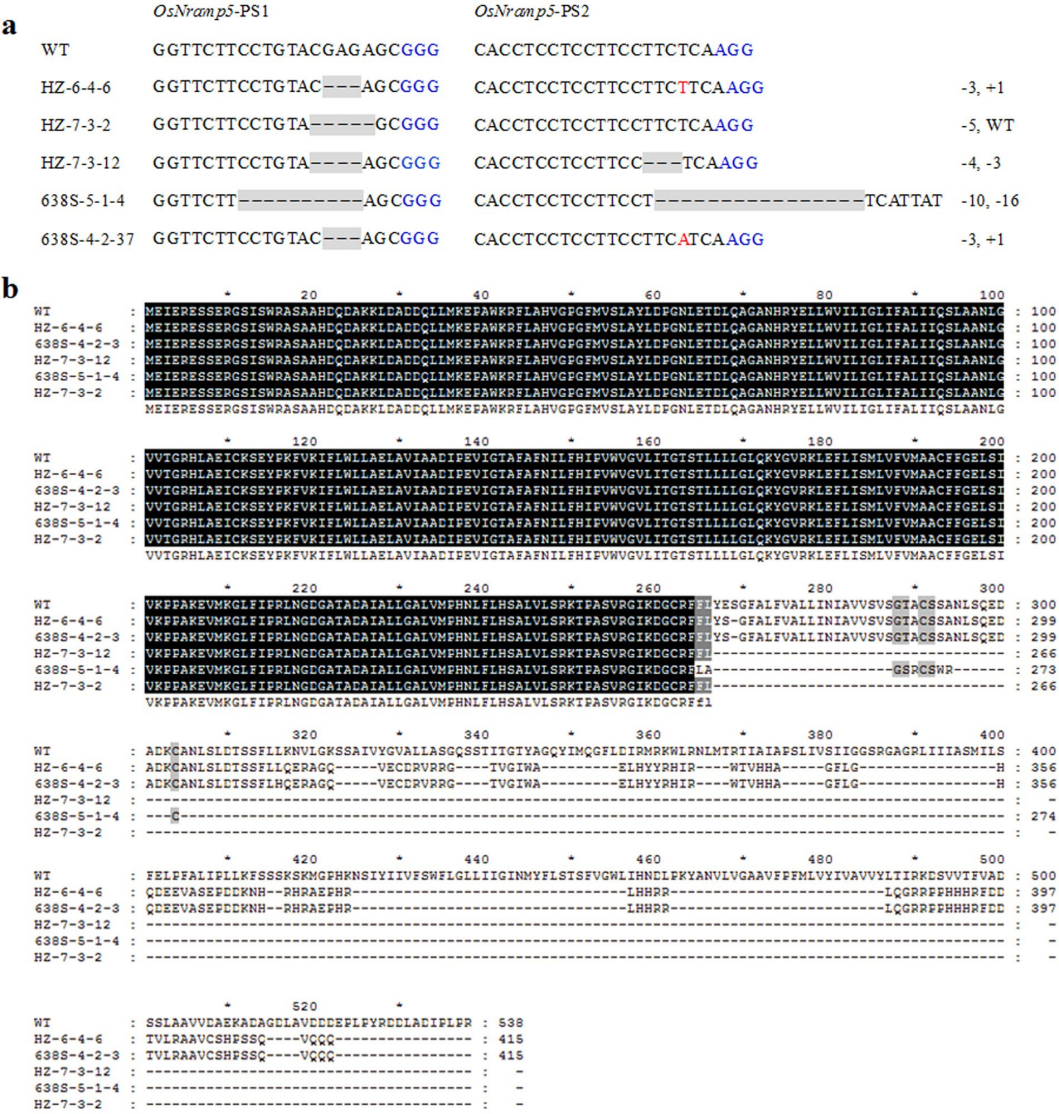
Homozygous *osnramp5* mutants induced by CRISPR/Cas9. **(a)** DNA sequence alignments for the 5 homozygous *osnramp5* mutants identified in the T_1_ generation, together with a wild-type (WT) control. The numbers on the right side are the sizes of the indels, with “−” and “+” showing deletion and insertion of nucleotides involved, respectively. **(b)** Deduced OsNramp5 amino acid sequence alignments for the 5 homozygous mutants and WT. Each of the mutant alleles codes for truncated and disrupted OsNramp5 proteins.

We also tracked the segregation of transgene (T-DNA) in the T_1_ population of the 4 T_0_ plants (Supplementary Table 2), based on a PCR assay of *Cas9* and *HPT*, which are the elements of T-DNA (Supplementary Fig. 1). The absence of transgenes was determined by negative PCR results of both *Cas9* and *HPT*. Transgene-free plants were observed in the progeny of all detected T_0_ plants, with the proportion ranging from 13.6% to 35.0% (Supplementary Table 2). These results indicated that transgene-free homozygous mutants could be easily obtained in the T_1_ generation, as the inheritance of T-DNA and the targeted gene was relatively independent. We isolated 5 transgene-free homozygous knockout lines with coding frame shifts and premature translational stops, including HZ-6-4-6, HZ-7-3-2, HZ-7-3-12, 638S-5-1-4, and 638S-4-2-37, in the T_1_ generation to produce the T_2_ population (Fig. 2).

### Effect of knocking out OsNramp5 on metal accumulation and major agronomic traits in *indica* rice

**Figure 3:**
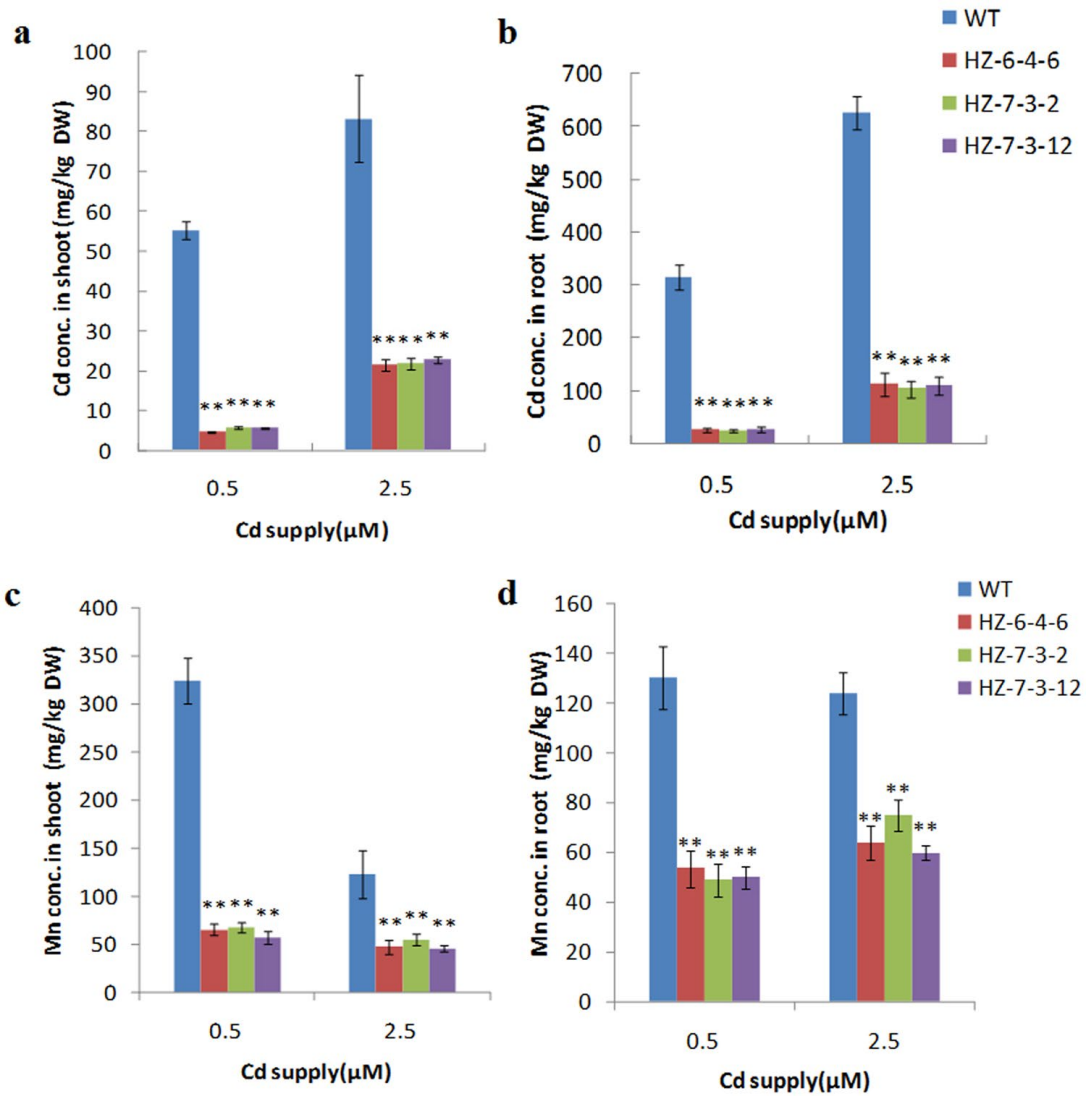
Concentrations of Cd and Mn in shoots and roots at different Cd levels. Three *osnramp5* mutant lines (HZ-6-4-6, HZ-7-3-2, HZ-7-3-12) and WT plants (HZ) were grown in normal nutrient solution for 2 weeks and then transferred to the nutrient solution containing 0.5 or 2.5 µM Cd for another 2 weeks. The shoots and roots were harvested and subjected to mineral analysis by inductively coupled plasma optical emission spectrometry (ICP-OES). **(a** and **b)** Cd concentrations in the shoots **(a)** and roots **(b)**. **(c** and **d)** Mn concentrations in the shoots **(c)** and roots **(d)**. DW, dry weight. Data are means ± SD of three biological replicates, and three plants were mixed in one replication for metal determination. Two asterisks indicate statistically significant difference in comparison to WT at *P* < 0.01 by Student’s *t* test.

**Figure 4:**
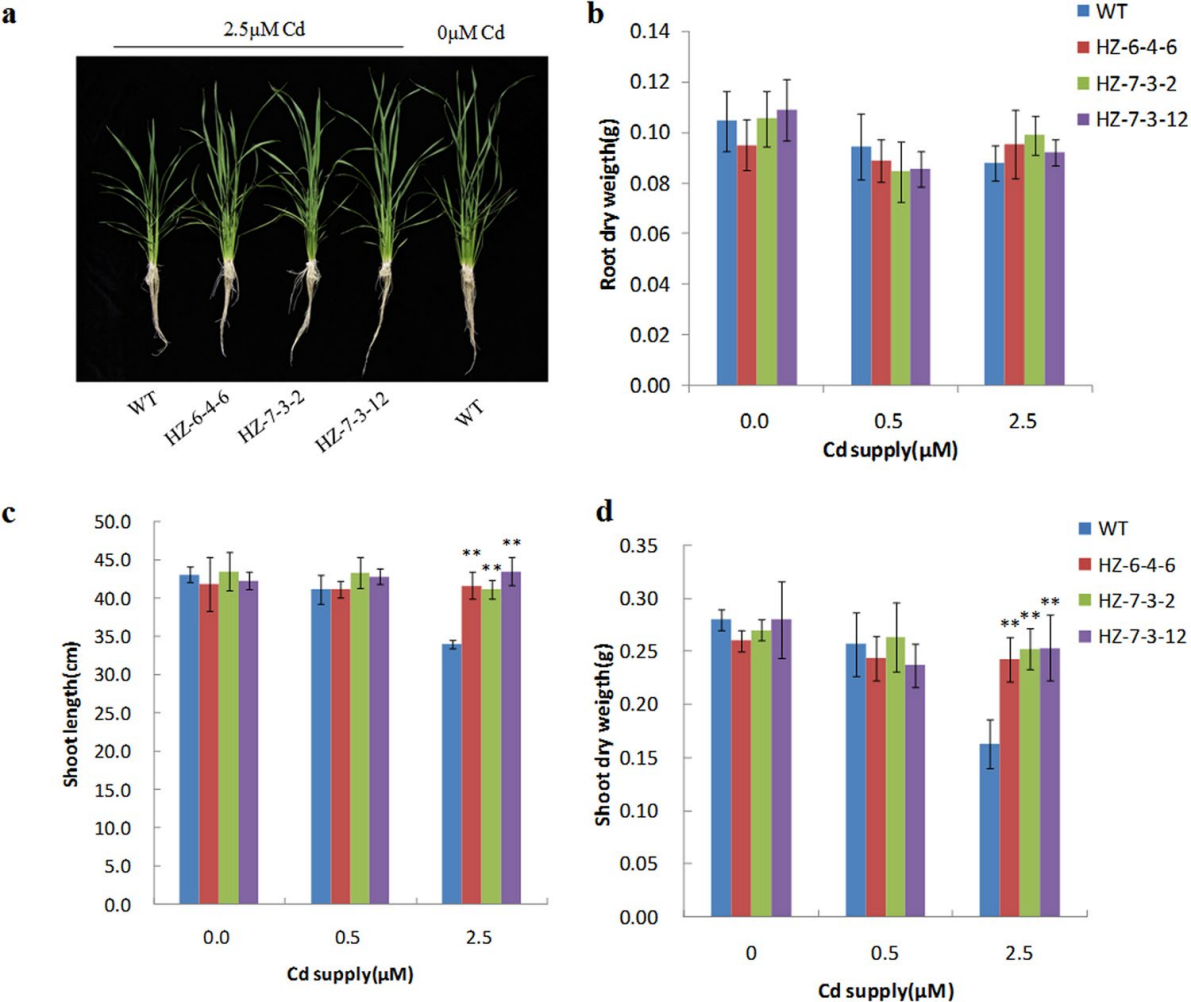
Phenotypic analysis of *osnramp5* mutants at different Cd levels. Three mutant lines (HZ-6-4-6, HZ-7-3-2, HZ-7-3-12) and WT plants (HZ) were cultivated in normal nutrient solution for 2 weeks and then transferred to the nutrient solution containing 0.5 or 2.5 µM Cd for another 2 weeks. **(a)** Plant morphologies of the mutant lines and WT in response to 2.5 µM Cd exposure. **(b)** Root dry weight. **(c)** Shoot length. **(d)** Root dry weight. Data are means ± SD of three biological replicates, and three plants were mixed in one replication. Two asterisks indicate statistically significant difference in comparison to WT at *P* < 0.01 by Student’s *t* test.

To measure metal accumulation and agronomic traits in the *OsNramp5* knockout mutants, the progeny of HZ-6-4-6, HZ-7-3-2, HZ-7-3-12, and WT plants were cultivated in nutrient solution supplemented with Cd or in soil contaminated by Cd. Metal analysis of seedlings grown in hydroponic culture with 0.5 µM or 2.5 µM Cd showed that both the Cd and Mn concentration in shoots and roots was much lower in *osnramp5* mutant lines than in WT plants (3.7-13 fold difference of Cd and 1.7–5.6 fold difference of Mn; Fig. 3). Under the toxic condition of 2.5 µM Cd, growth of WT plants was severely inhibited compared with that under no Cd stress, whereas growth of mutant lines was close to that of WT plants under no Cd stress (Fig. 4), indicating that the decrease of Cd in mutants could greatly rescue the reduced growth in the presence of normal-level Mn. In contrast, the concentration of other relevant metals in roots, including iron (Fe), zinc (Zn) and copper (Cu), was not different between the mutants and WT plants (Supplementary Fig. 2d,e,f). In shoots of mutants, the Zn concentration remained essentially unchanged, while the Fe concentration was lower and the Cu concentration was higher compared with that in shoots of WT plants (Supplementary Fig. 2a,b,c). However, the change in concentrations of Fe and Cu was much less than that for Cd and Mn.

Mn concentration was mostly decreased among essential micronutrients in *osnramp5* mutants, and the great reduction in Mn concentration may affect plant growth. To gain insight into the effect of low Mn supply to growth of *osnramp5* mutants, both mutant and WT plants were grown in normal hydroponic culture for 12 d, and then transferred to nutrient solutions with different low concentrations of Mn for 18 d. Under the condition of no Mn supply, the mutant lines showed severely stunted growth and yellowing leaves (typical symptoms of Mn deficiency), in contrast to the phenotypes of WT plants. However, the mutants grew similarly to WT plants in the presence of 2 µM or higher Mn (Supplementary Fig. 3). These results imply that mutants are tolerant of low Mn conditions to a certain extent.

**Figure 5:**
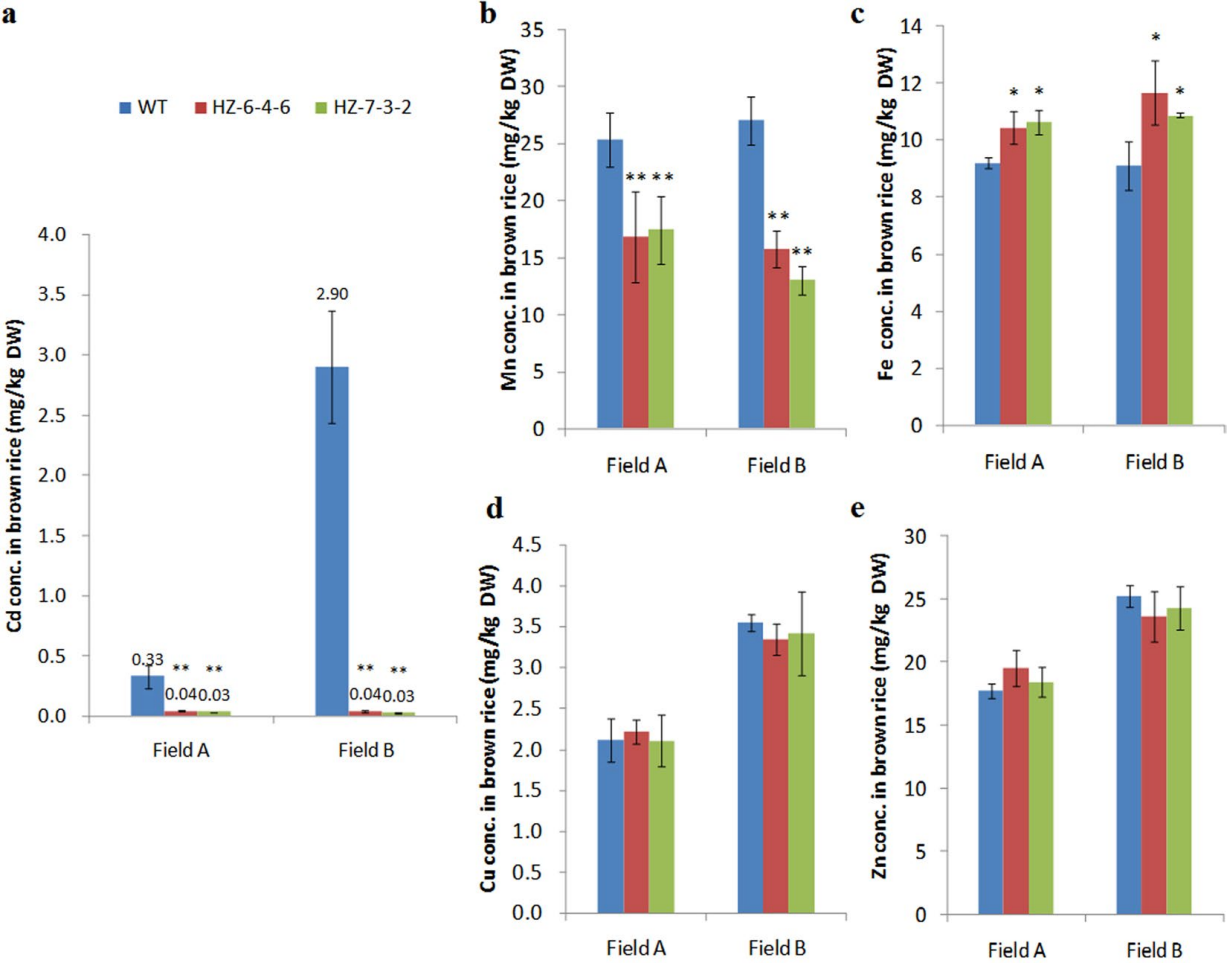
Metal concentrations in the brown rice of *osnramp5* mutants. Two mutant lines (HZ-6-4-6, HZ-7-3-2) and WT plants (HZ) were cultivated together in two restricted experimental paddy fields containing 1.69 mg/kg (Field A) and 2.37 mg/kg (Field B) Cd in the soil, respectively. **(a)** Cd, **(b)** Mn, **(c)** Fe, **(d)** Cu and **(e)** Zn concentrations in brown rice were determined by ICP-OES. DW, dry weight. Data are means ± SD of three biological replicates, and grains of three plants were mixed in one replication. One or two asterisks indicate statistically significant difference in comparison to WT at *P* < 0.05 or *P* < 0.01 by Student’s *t* test.

When mutant plants were grown until maturity in two restricted experimental paddy fields contaminated by different Cd levels (1.69 mg/kg and 2.37 mg/kg), the Cd concentration in grains (brown rice) was <0.05 mg/kg, compared with an average of 0.33 mg/kg and 2.90 mg/kg in the grains of WT plants (Fig. 5a). Similar results were found in the pot with Cd-polluted soil, where the Cd concentration in grains of mutants was also <0.05 mg/kg, and that of WT was 1.06 mg/kg (Supplementary Fig. 4). These results suggest that knockout of *OsNramp5* could produce low Cd-accumulating rice in high Cd-polluted fields. The analysis of other relevant metals in grains showed that mutants accumulate 34–53% less Mn than in WT plants (Fig. 5b). Unexpectedly, the concentration of Fe was higher in the mutants (Fig. 5c), but no significant differences in Cu or Zn concentration were observed between mutants and WT plants (Fig. 5d and e). In straw, the concentrations of Cd and Mn were much lower in the mutants than in WT plants, and the concentrations of Fe, Zn, and Cu were similar between the mutants and WT plants (Supplementary Table 3 and Supplementary Table 4).

**Figure 6:**
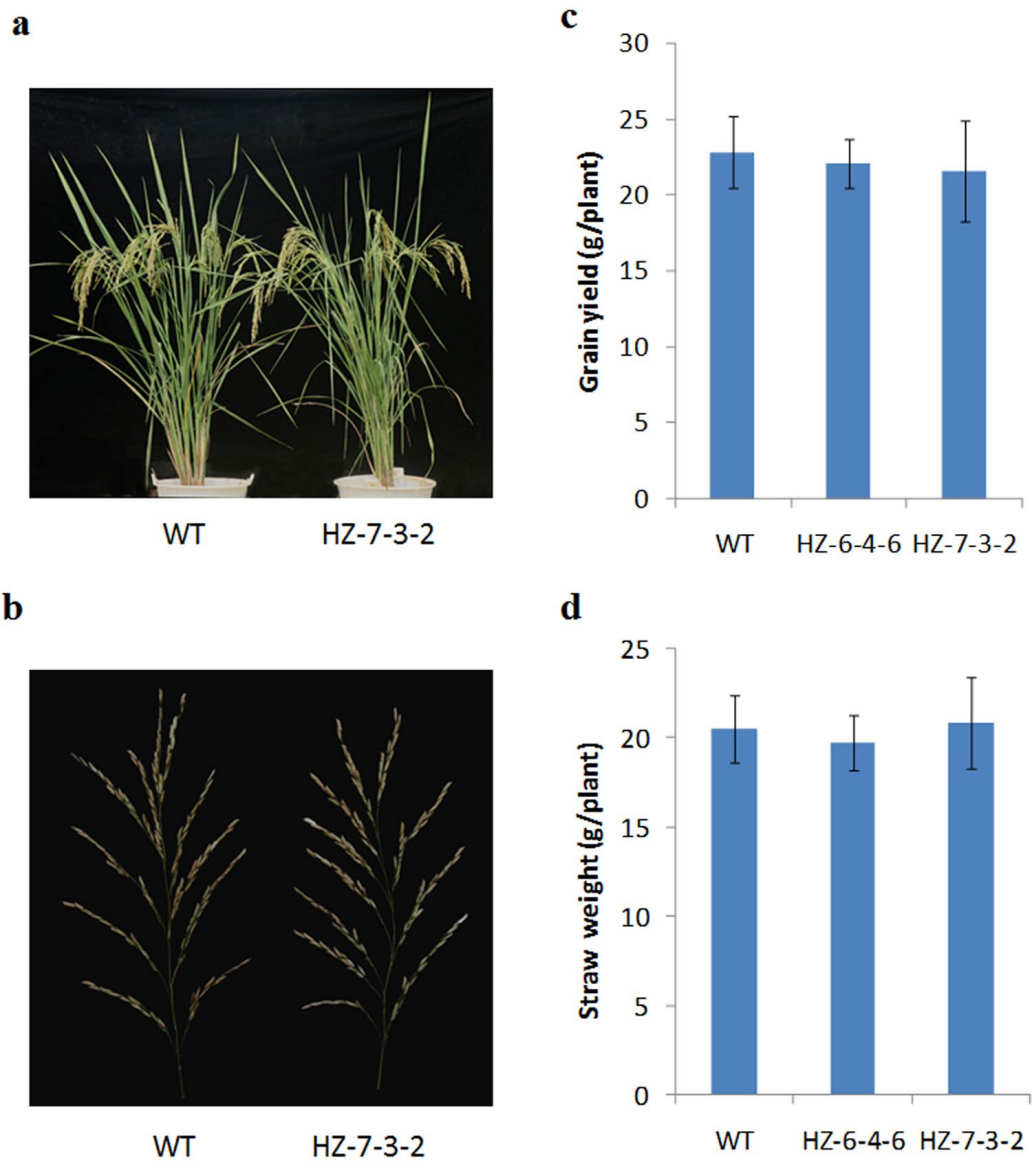
Morphology and yield analysis of *osnramp5* mutants. Two mutant lines (HZ-6-4-6, HZ-7-3-2) and WT plants (HZ) were cultivated in the Cd contaminated experimental field (Field A). **(a)** Plant morphology. **(b)** Panicle morphology. **(c)** Grain yield. **(d)** Straw dry weight. There is no significant difference between WT and mutant lines in grain yield and straw dry weight according to Student’s *t* test.

Phenotypic analysis showed that there were no visible differences in plant growth or panicle morphologies between the mutant lines and the WT plants (Fig. 6a and b; Supplementary Fig. 5). Moreover, mutant lines exhibited no significant differences in grain yield, straw weight (Fig. 6c and d), grain quality (Supplementary Table 5), or other main agronomic traits (Supplementary Table 6), compared with WT plants. These results demonstrate that knocking out *OsNramp5* could have negligible effects on growth, biomass, and main agronomic traits, under our cultivation conditions.

### Development of promising hybrid rice lines with extremely low Cd content in grains

**Figure 7:**
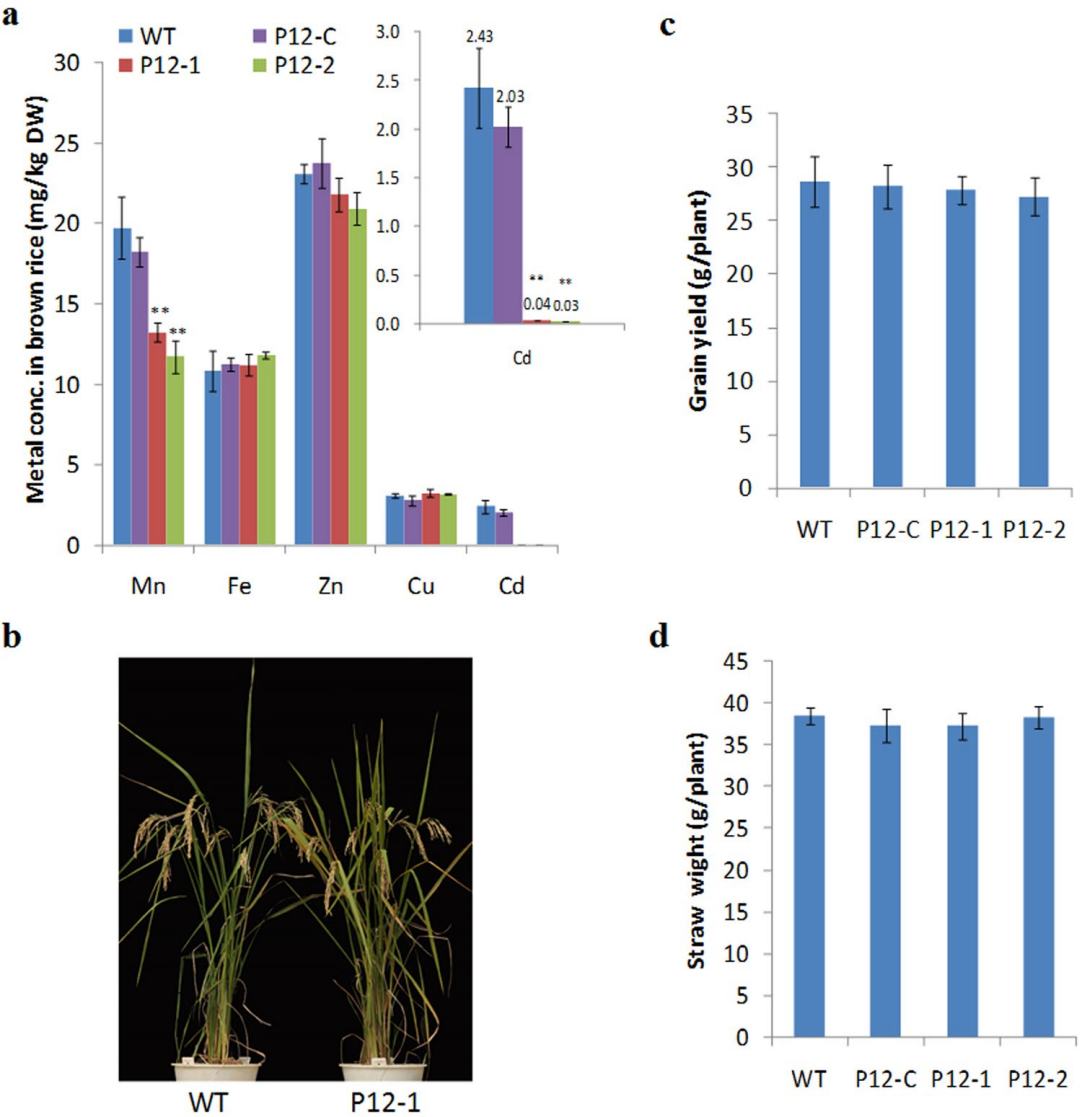
Morphology and yield analysis of mutant hybrid rice lines. Two *osnramp5* mutant hybrid rice lines (P12-1, P12-2), control line (P12 C) and WT plants (LLYHZ) were cultivated in the highly Cd-contaminatedexperimental field (Field B). **(a)** Metal concentration in brown rice. **(b)** Plant morphology. **(c)** Grain yield. **(d)** Straw dry weigh. DW, dry weight. Two asterisks indicate statistically significant difference in comparison to WT at *P* < 0.01 by Student’s *t* test.

We developed two independent mutant hybrid rice lines (P12-1, P12-2) by crossing the 638S mutants (638S-5-1-4, 638S-4-2-37) and HZ mutants (HZ-6-4-6, HZ-7-3-12), respectively. The hybrid rice line P12-C generated by crossing WT 638S and HZ mutant (HZ-6-4-6) served as a control. These mutants and WT LLYHZ plants were grown in the experimental paddy field with 2.37 mg/kg Cd in soil. Metal analysis showed that Cd concentration in grains of mutant lines (P12-1, P12-2) was decreased by more than 98% (<0.05 mg/kg) and the Mn concentration was reduced by 41% compared with WT plants. However, there was no significant difference in the concentrations of Fe, Zn, or Cu in grains between the mutant lines (P12-1, P12-2) and WT plants (Fig. 7a). In contrast, concentrations of all five metals in the grains of P12-C were similar to that of WT plants (Fig. 7a), suggesting that metal content was changed only when both of the parental lines were mutated. In addition, there was no obvious difference in plant morphology, grain yield, or straw weight between the mutant lines and WT plants (Fig. 7b–d). These results demonstrate that the knockout of *OsNramp*5 by the CRISPR/Cas9 system could be applied to two-line *indica* hybrid breeding, developing Cd pollution-safe hybrids suitable for planting in high Cd paddy fields.

## Discussion

There is a worldwide concern about Cd contamination in rice grains, and thus lowering grain Cd content is critical, but still challenging. In the present study, *OsNramp5* was knocked out using the CRISPR/Cas9 system, in the popular male sterile line 638S and restorer line HZ of *indica* hybrid rice. In addition, the mutant hybrid LLYHZ was developed by crossing the mutant parental lines. The *osnramp5* mutants and their hybrids had nearly undetectable levels of Cd in the grains, and yield and main agronomic traits were not affected when they were grown in Cd-contaminated paddy fields. These phenotypes were similar to that of the *osnramp5* mutant with the Koshihikari background^21^. In contrast, the growth and yield of the T-DNA insertion mutant of *OsNramp5* with Zhonghua 11 background were heavily influenced^20^. The inconsistent effect of *OsNramp*5 mutation on plant yield probably arises from different Mn concentration in mutants caused by differences in mutation sites, genetic backgrounds, or supplies of Mn.

Mn is an essential micronutrient for plant growth and development, and thus Mn deficiency could result in lower yield in *osnramp5*. Mn concentrations in the shoots/straws were much higher in HZ and LLYHZ knockout mutants than in the Zhonghua11 knockout mutant, in both normal hydroponic culture and soil culture (Fig. 3c, Supplementary Table 3 and Supplementary Table 4)^20^. In addition, Mn concentrations in straws of HZ and LLYHZ knockout mutants grown in the paddy fields were decreased by 80.6–82.6% compared with WT plants, and were either higher than or close to 150 mg/kg (Supplementary Table 3 and Supplementary Table 4). This level of Mn concentration was high enough to cause toxic symptoms in barley^48^. In contrast, the Mn concentration in straw of the Zhonghua 11 knockout mutant grown in soil culture was approximately 95% less than that in WT plants^20^. On the other hand, the HZ knockout mutants probably require less Mn for normal growth than the Zhonghua 11 knockout mutant. Here, we performed hydroponic culture with a method similar to that used by Yang *et al*.^49^, except with a different Mn concentration gradient. Mn concentration in the shoots of the HZ knockout mutant (2 µM Mn supplied) was close to that in the Zhonghua 11 knockout mutant (1.6 µM Mn supplied) (Supplementary Table 7)^49^. However, the HZ knockout mutant had a similar phenotype to WT plants, whereas the Zhonghua 11 knockout mutant showed reduced growth (Supplementary Fig. 3)^49^. It has been described that the critical deficiency concentrations of Mn in plants are similar, varying between 10 and 20 mg/kg dry weight in fully expanded leaves^50^. Mn concentrations in the shoots of both the HZ knockout mutant and Zhonghua 11 knockout mutant were within this range. It is likely that the higher Mn concentration in straws of HZ knockout mutants and their lower sensitivity to low-Mn concentration, caused the different influence on their yield compared with that of Zhonghua11 knockout mutant.

In contrast to other crops, rice is usually cultivated under flooded conditions, where is anaerobic and Mn concentration is generally high. Rice has developed strategies to accumulate high Mn without symptoms of toxicity^48^. It is possible that the growth of *osnramp5* mutants would not be inhibited, when mutants were cultivated in Cd-polluted soils with a low Mn concentration, by applying Mn fertilizer or prolonging flooded time properly, due to greatly increased bioavailability of Mn under flooded conditions^51^. The effects of knocking out *OsNramp5* on Mn concentration in shoots, on plant growth, and on main agronomic traits under gradient low-Mn field conditions need to be studied further in various rice cultivars with multiple genetic backgrounds.

The markedly lower accumulation of Mn and Cd in the *osnramp5* mutant in the present study supports the crucial role of OsNramp5 for Mn and Cd uptake^20,52^. In addition, increasing Cd in the external solution decreased Mn accumulation in shoots of WT plants, while this effect was not obvious in *osnramp5* mutants (Fig. 3c). This result implies that Mn and Cd compete for uptake mediated by the OsNramp5 transporter, and an abundance of one metal could disturb the uptake of the other. Rice is a staple food crop with high Mn accumulation, and the majority of Mn is taken up by OsNramp5^20^. It seems likely that external Cd competitively enters the root cells during the process of OsNramp5 mediating uptake of Mn from the soil, as OsNramp5 has limited substrate specificity. Genotypic variation of Cd accumulation between two rice subspecies, *japonica* and *indica*, has been reported^11–14^, but little is known about the molecular mechanism. As OsNramp5 contributes greatly to Cd accumulation in rice, further analysis is needed to determine whether there is a difference in the expression level of *OsNramp5* between *indica* and *japonica* rice, and whether there is a positive correlation between the expression level of *OsNramp5* and the Cd content in rice.

It has been reported that knockdown of *OsNramp5* could lead to more Cd accumulation in shoots of RNAi lines than WT plants, making it a promising candidate for phytoremediation in paddy fields^52,53^. However, *OsNramp5* RNAi knockdown lines generated by Sasaki *et al*.^20^, as well as *OsNramp5* knockout lines in our study and other previous studies^20,21,49^, accumulated much less Cd in shoots than WT plants. This contradictory effect is not likely to arise from the direct role of OsNramp5. There is strong evidence that Cd and Fe share transporters in rice, such as *OsIRT1, OsIRT2, OsNramp1*, and *OsNramp5* ^20,22,23^. The expression of some Fe/Cd transporter genes were highly induced in *OsNramp5* knockout or knockdown lines, especially under the condition of Fe (II) deficiency^49,52,53^. The increased expression of these Fe/Cd transporters enhanced Cd translocation from root to shoot^22,52^. The expression of *OsNramp5* in Anjana Dhan RNAi lines was suppressed only by 1/3 to 1/2 of that in WT plants^53^, so the decreased amount of Cd in shoots by suppression of *OsNramp5* would be less than the increased amount of Cd by induction of other Fe/Cd transporters, giving rise to higher Cd concentrations in shoots of RNAi lines. But the degree of decrease in Cd amount in shoots was very serious, when the structure of OsNramp5 protein was broken down in knockout lines^21^, or when the expression of *OsNramp5* was extremely suppressed in knockdown lines^20^, which cannot be complemented by an increase in Cd translocation through other Fe/Cd transporters.

In conclusion, we evaluated the effects of *OsNramp5* mutation on accumulation of Cd and relevant metals, and on main agronomic traits in *indica* rice using CRISPR/Cas9-mediated mutagenesis. The high Cd accumulation trait of *indica* rice was modified quickly, effectively, and conveniently, whereas yield and main agronomic traits were not impaired. Transgenes were segregated out through selfing, creating genome-edited mutant plants that were free of transgenes. These plants were not different from plants carrying spontaneous mutations or mutations induced by physical and chemical treatment, in addition to the advantage that the precise mutation reduced the risk of introducing undesired traits^41,54^. Thus, these genome-edited mutants could circumvent public and governmental concerns about introduced persistent transgenes or other unwanted genomic fragments. Our study developed promising *indica* rice lines and their hybrids with extremely low Cd content in grains and without compromising yield using the CRISPR/Cas9 mediated *OsNramp5* editing system. This approach can be used to reduce grain Cd levels in other widespread *indica* rice cultivars, facilitating the development of precision breeding targeted at Cd pollution-safe rice.

## Methods

### Vector construction and rice transformation

The Cas9 plant expression vector (pYLCRISPR/Cas9Pubi-H) and sgRNA expression vector (pYLgRNA) were provided by Prof. Yao-Guang Liu (South China Agricultural University). According to the design principles of the target sequences in the CRISPR/Cas9 system, 19 to 20 bases upstream of the PAM motif were selected as candidate target sequences. A BLAST search (http://blast.ncbi.nlm.nih.gov/Blast.cgi) of the target sequences (including PAM) against the rice genome was carried out to confirm their targeting specificity in the genome. CRISPR/Cas9 plant expression vectors were constructed as previously described^45^, based on Golden Gate cloning. Binary constructs were then introduced into *Agrobacterium tumefaciens* strain EHA105. Popular parental lines of *indica* hybrid rice (*Oryza sativa* L. cv. Huazhan and Longke 638S) were used for plant transformation. *Agrobacterium*-mediated transformations of embryogenic calli were performed by Wuhan Biorun biological technology Co., Ltd. After 4 weeks of rooting, regenerated rice plants were transferred to plastic buckets in a greenhouse maintained at 30 °C during the day and 26 °C at night.

### Mutation detection and assay of transgene-free plant lines

Genomic DNA was extracted from at least 5 leaves of different tillers during the rice maturity period using the cetyltrimethyl ammonium bromide (CTAB) method^55^. PCR was performed to amplify the genomic region containing the CRISPR/Cas9 target sites using specific primers (Supplementary Table 8). The PCR products were purified and directly sequenced. If the sample produced superimposed sequence chromatograms, it was cloned into the pEASY-Blunt (Trans Gen Biotech, Beijing, China), and at least 10 clones were sequenced to identify the genotype of the mutation. Multiple amino acid sequence alignments were performed using ClustalX2^56^ and displayed using GeneDoc software (http://www.softpedia.com/get/Science-CAD/GeneDoc.shtml#download).

The identification of transgene-free plants was conducted using T_1_ generation plants. The plants were analysed by PCR using *HPT*-specific and *Cas9*-specific primers (Supplementary Table 8) and agarose gel electrophoresis. The CRISPR/Cas9 plasmid targeting *OsNramp5* and the T_0_ transgenic plants were selected as positive controls, and HZ or 638S and H_2_O were used as negative controls. *HPT*- and *Cas9*-negative plants were considered transgene-free plants.

### Hydroponic experiments

Seedlings of three independent mutant lines and WT plants were grown hydroponically in rice culture solution described by Yoshida *et al*.^57^. The plants were grown in a greenhouse at 26 °C to 30 °C under natural light. The culture solution was renewed every 3 d. In the Cd treatment experiments, plants were cultivated in normal nutrient solution for 14 d and then transferred to the nutrient solution containing 0.5 or 2.5 µM Cd (added as CdCl_2_) for another 14 d. The roots were washed three times with deionized water and separated from the shoots, and subjected to metal concentration determination as described below. In the low Mn concentration gradient experiments, plants were grown in normal nutrient solution for 12 d, and then transferred to the nutrient solution with 0, 2, 4, or 6 µM Mn (lower than normal culture) for 18 d. Three biological replicates were used for each treatment.

### Field and pot experiments

The independent mutant lines and WT plants were cultivated in different Cd-polluted experimental paddy fields in China. The soil Cd concentrations were 1.69 mg/kg in field A, 2.37 mg/kg in field B and 1.53 mg/kg in field C; the soil Mn concentrations were 246 mg/kg in field A, 162 mg/kg in field B and 849 mg/kg in field C; and the soil pH values were 5.1 in field A, 5.5 in field B and 6.1 in field C. 28-day-old seedlings were transplanted into the flooded field plots, with one plant per hill, spaced at 20 cm × 20 cm. The plot for each line included 4 rows, with a total of 40 plants. The fields were irrigated intermittently until grain maturity. We applied inorganic fertilizers containing N, K, and P using standard methods. After grains were harvested, metal concentration and major agronomic traits, including grain yields and straw weight, were analysed. Rice quality was measured using the standard of NY/T 593-2013 published by the Rice Product Quality Supervision and Inspection Centre, Ministry of Agriculture, China (http://www.zbgb.org/27/StandardDetail1476335.htm). The field experiments were arranged in a randomized complete block design with three plot replications. In pot experiments, 28-day-old seedlings were transplanted into plastic pots filled with polluted paddy soil with 1.31 mg/kg Cd and 259 mg/kg Mn concentration, with a pH value of 5.7. Plants were grown in a greenhouse at 26 to 30 °C under natural light, with three biological replicates. Tap water was supplied intermittently throughout the cultivation.

### Metal concentration determination

Shoots, roots, and brown rice samples were harvested and dried at 70 °C for 2 d. The dried samples were ground into powder and digested with an acid mixture of HNO_3_-HClO_4_ (6:1,v/v), and then placed on an electric heating plate for digestion until they were almost completely evaporated. After cooling, the residues were dissolved in 1% HNO_3_ (1:10, v/v) and filtered. The filtered solutions were diluted to 10 mL for brown rice samples, and 25 mL for shoot and root samples. Then, the metal concentration was determined by inductively coupled plasma optical emission spectrometry (SPS3 ICP-720 OES; Agilent Technologies) at the following wavelengths: 226.502 (Cd), 293.305 (Mn), 238.204 (Fe), 206.200 (Zn), and 324.754 (Cu) nm.

## Electronic supplementary material

Supplementary Information
