# Supplementary material for: Knockout of OsNramp5 using the CRISPR/Cas9 system produces low Cd-accumulating indica rice without compromising yield
Source: Sci Rep. 2017 Oct 31;7:14438. doi: 10.1038/s41598-017-14832-9 (PMC5663754; doi:10.1038/s41598-017-14832-9)
Supplement: Supplementary file 1 — Supplementary Information [file 41598_2017_14832_MOESM1_ESM.pdf]

Knockout of *OsNramp5* using the CRISPR/Cas9 system produces low Cd-accumulating *indica* rice without compromising yield

Li Tang[1,2], Bigang Mao[2], Yaokui Li[2], Qiming Lv[2], LiPing Zhang[3], Caiyan Chen[3], Hanjie He[2,4], Weiping Wang[2], Xiongfeng Zeng[1], Ye Shao[2], Yinlin Pan[2], Yuanyi Hu[2],Yan Peng[2], Xiqin Fu[2], Hongqing Li[5*], Shitou Xia[1*] & Bingran Zhao[2*]

Includes

Supplementary Figure 1. Detection of T-DNA fragments in T_1 mutants using PCR and gel electrophoresis.

Supplementary Figure 2. Metal concentrations of *osnramp5* mutants.

Supplementary Figure 3. Phenotypes of *osnramp5* mutants in response to low Mn supply.

Supplementary Figure 4. Mineral concentrations in brown rice of *osnramp5* mutants.

Supplementary Figure 5. Plant morphology of *osnramp5* mutants in seedling stage and heading stage.

Supplementary Table 1. Mutation in the putative off-target sites of CRISPR/Cas9.

Supplementary Table 2. Inheritance of mutations induced by CRISPR/Cas9 from T_0 generation to T_1 generation.

Supplementary Table 3. Metal concentration in straw of mutant plants grown in Cd-polluted paddy field (field B).

Supplementary Table 4. Metal concentration in straw of mutant plants grown in Cd-polluted paddy field (field C).

Supplementary Table 5. Grain quality traits of *osnramp5* mutants.

Supplementary Table 6. Major agronomic traits of *osnramp5* mutants.

Supplementary Table 7. Mn concentration in shoots and roots of mutant plants grown in hydroponic culture with low Mn supply.

Supplementary Table 8. Primers used for sequencing and transgenic analysis.

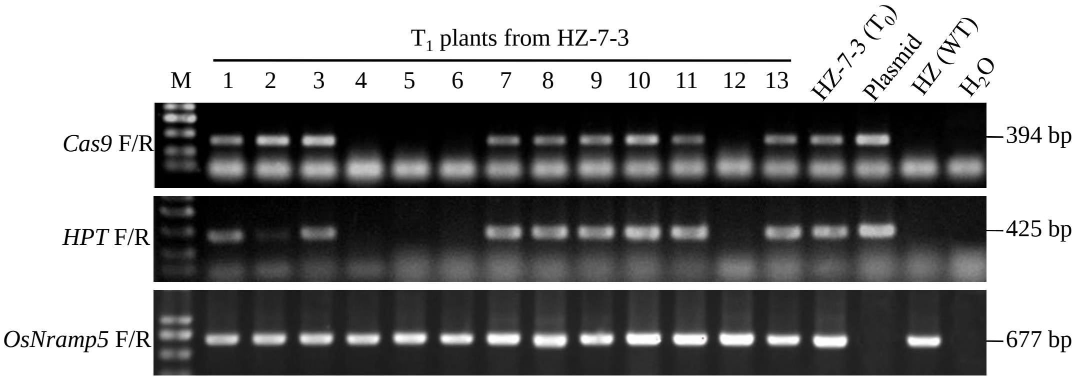

Supplementary Figure 1. Detection of T-DNA fragments in T_1 mutants using PCR and gel electrophoresis. PCR product amplified from the target region in *OsNramp5* is used as an internal control in each reaction. M refers to the DNA size marker. The T_0 plants and CRISPR/Cas9 vectors targeting *OsNramp5* are positive controls; the wild-type plants and H_2O are negative controls. The numbers on the right of the gel image indicate the expected sizes of the amplicons.

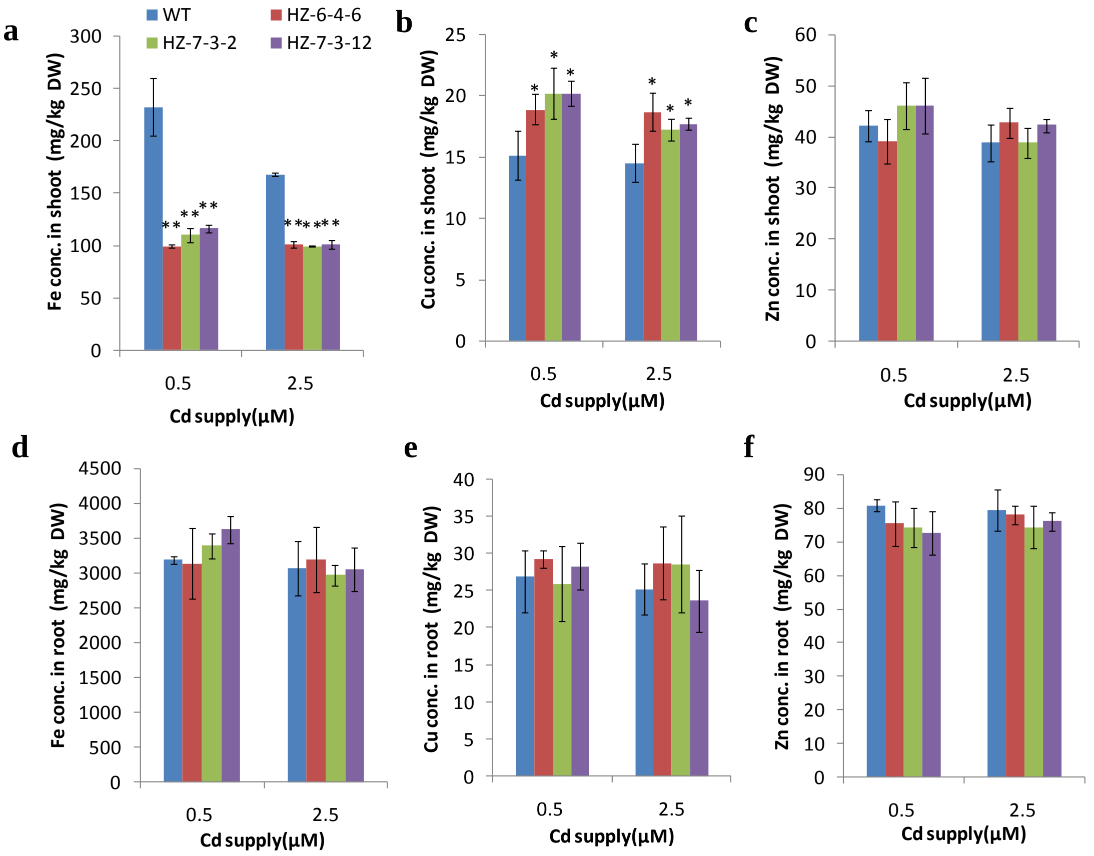

Supplementary Figure 2. Metal concentrations of *osnramp5* mutants. Three mutant lines (HZ-6-4-6, HZ-7-3-2, HZ-7-3-12) and WT plants (HZ) were cultivated in normal nutrient solution for 2 weeks and then transferred to the nutrient solution containing 0.5 or 2.5 μM Cd for another 2 weeks. **(a and d)** Fe concentrations in the shoots **(a)** and roots **(d)**. **(b and e)** Cu concentrations in the shoots **(b)** and roots **(e)**. **(c and f)** Zn concentrations in the shoots **(c)** and roots **(f)**. DW, dry weight. Data are means ± SD of three biological replicates, and three plants were mixed in one replication for metal determination. One or two asterisks indicate statistically significant difference in comparison to WT at $P < 0.05$ or $P < 0.01$ by Student's t test.

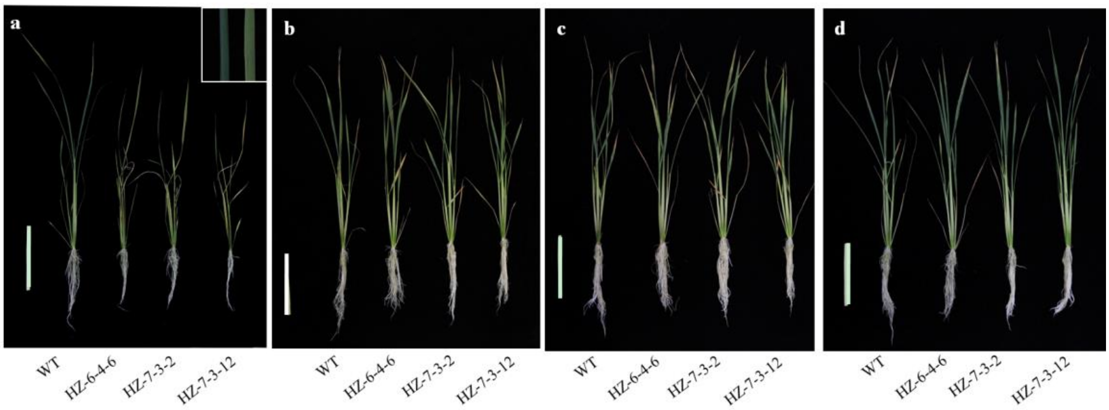

Supplementary Figure 3. Phenotypes of *osnramp5* mutants in response to low Mn supply. Three mutant lines (HZ-6-4-6, HZ-7-3-2, HZ-7-3-12) and WT plants (HZ) were grown in normal nutrient solution for 12 d, and then transferred to the nutrient solution with 0 **(a)**, 2 **(b)**, 4 **(c)** or 6 **(d)** μM Mn (lower than normal culture) for 18 d. The youngest leaves of WT (left) and HZ-6-4-6 (right) grown in absence of Mn are shown in the top right corner of **(a)**. Scale bars, 20 cm.

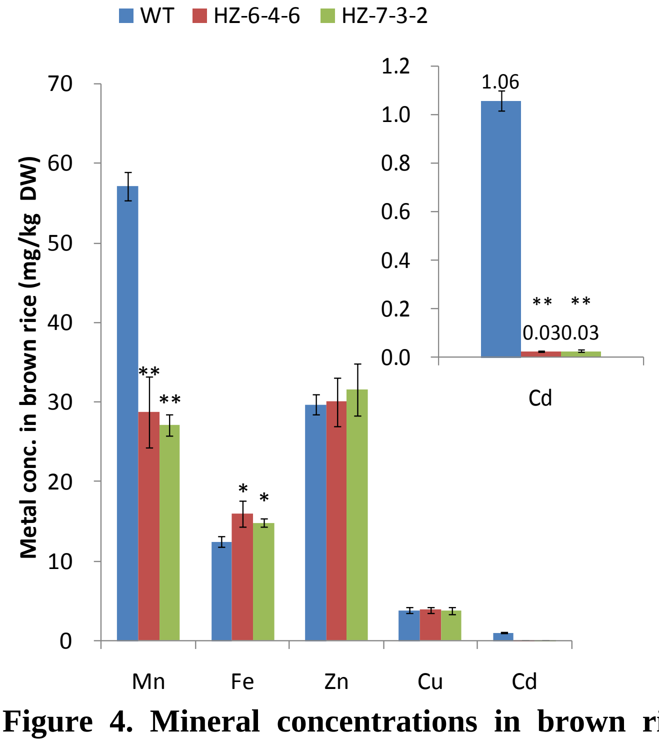

Supplementary Figure 4. Mineral concentrations in brown rice of *osnramp5* mutants. Two mutant lines (HZ-6-4-6, HZ-7-3-2) and WT plants (HZ) were grown in plastic pots filled with 1.31 mg/kg Cd polluted soil. DW, dry weight. Data are means ± SD of three biological replicates, and grains of two plants were mixed in one replication. One or two asterisks indicate statistically significant difference in comparison to WT at $P < 0.05$ or $P < 0.01$ by Student's t test.

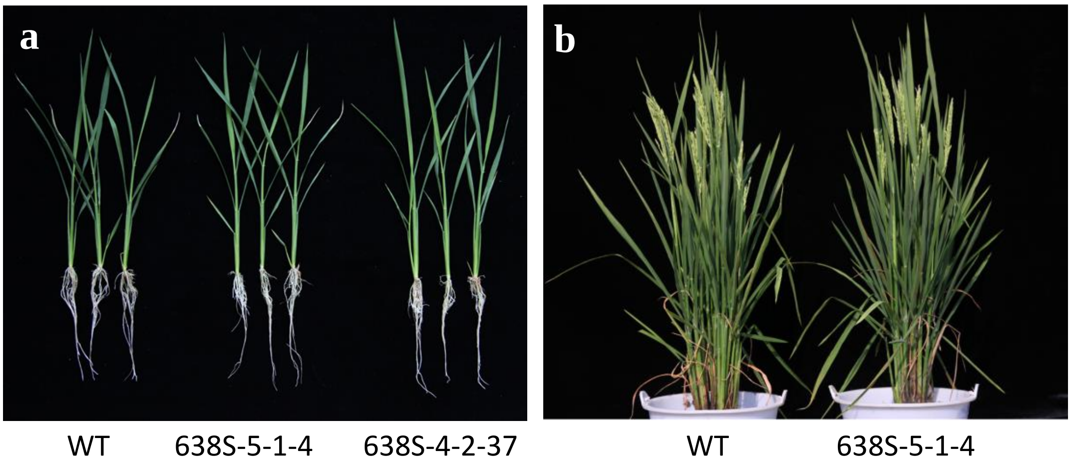

Supplementary Figure 5. Plant morphology of *osnramp5* mutants in seedling stage and heading stage.

(a) The mutant plants (638S-5-1-4 and 638S-4-2-37) and WT plants (638S) grown in nutrition solution for 20d. **(b)** The mutant plants and WT plants cultivated in experimental paddy field (Field A).

Supplementary Table 1. Mutations in the putative off-target sites of CRISPR/Cas9.

Target site	Putative off-target site	Putative off-target locus	Sequence of putative off-target site	No. of mismatching bases	No. of plants detected	No. of plants with mutations
OsNramp5-PS1	PS1-OS1	Chr5: 8315283-8315305	TCTTCTTCCTGTAAGAGAGAGGG	4	57	0
	PS1-OS2	Chr7: 22346920-22346942	TGGTGGCGCTGTACGAGAGCGGG	6	57	0
OsNramp5-PS2	PS2-OS3	Chr11: 3712812-3712834	CGGCGTCTCCTTCCTCCTCAAGG	5	57	0
	PS2-OS4	Chr3: 8396291-8396313	TTTTTTCTCCTTCCTTCTCAAGG	5	57	0

Mismatching bases are marked in red. The sequences of the PAM are underlined.

Supplementary Table 2. Inheritance of mutations induced by CRISPR/Cas9 from T_0 generation to T_1 generation.

T_0 plant	Target site	T_0 genotype	T_1 genotype (No. of plants)	χ^2(1:2:1)	T_0 Inheritance pattern	Transgene-free ratio (%) in T_1
HZ-6-4	*OsNramp5*-PS1	-3	-3(20)	ND	Homozygote	35.0 (7/20)
	OsNramp5-PS2	+1	+1(20)	ND	Homozygote	
638S-5-1	*OsNramp5*-PS1	-10	-10(22)	ND	Homozygote	13.6 (3/22)
	OsNramp5-PS2	-16	-16(22)	ND	Homozygote	
HZ-7-3	*OsNramp5*-PS1	-4/-5	-4(8), -4/-5(21), -5(9)	0.474 (P > 0.05)	Biallelic mutant	21.1 (8/38)
	OsNramp5-PS2	-3/+1a/+1b/WT	-3(9), -3/WT(22), 3/+1c(1), +1a(2), WT(4)	ND	Chimera	
638S-4-2	*OsNramp5*-PS1	-3/-4	-3(10), -3/-4(20), -4(7)	0.730 (P > 0.05)	Biallelic mutant	24.3 (9/37)
	OsNramp5-PS2	+1a/+1b/+1c/WT	+1a(8), +1a/WT(21),+1b/WT(1), +1a/-2(1),WT(6)	ND	Chimera	

ND, not detected.

Supplementary Table 3. Metal concentration in straw of mutant plants grown in Cd-polluted paddy field (field B).

Line	Cd (mg/kg)	Mn (mg/kg)	Fe (mg/kg)	Zn (mg/kg)	Cu (mg/kg)
**WT**	3.32 ±0.79	782.63 ±109.35	124.22 ±21.99	20.41 ±1.05	2.96 ±0.51
**HZ-6-4-6**	0.13 ±0.09**	151.79 ±21.16**	129.46 ±19.83	20.62 ±1.01	3.07 ±0.18
**HZ-7-3-2**	0.12 ±0.04**	145.72 ±32.33**	140.65 ±27.41	18.99 ±1.64	3.31 ±0.59

Two mutant lines (HZ-6-4-6, HZ-7-3-2) and WT plants (HZ) were grown until maturity in the Cd contaminated experimental field (field B). Values shown are the means ± SD (n =3), and straws of three plants were mixed in one replication. Two asterisks indicate significant difference in comparison to WT at $P < 0.01$ by Student's *t* test.

Supplementary Table 4. Metal concentration in straw of mutant plants grown in Cd-polluted paddy field (field C).

Line	Cd (mg/kg DW)	Mn (mg/kg DW)	Fe (mg/kg DW)	Zn (mg/kg DW)	Cu (mg/kg DW)
**HZ-WT**	5.93 ±0.78	950.49 ±162.31	355.09 ±80.38	73.29 ±8.31	11.69 ±0.65
**HZ-6-4-6**	0.17 ±0.02**	165.34 ±26.04**	332.03 ±93.61	69.01 ±6.35	13.28 ±3.27
**HZ-7-3-2**	0.16 ±0.02**	170.43 ±26.96**	345.94 ±92.58	71.41 ±2.06	14.41 ±3.95

Two mutant lines (HZ-6-4-6, HZ-7-3-2) and WT plants (HZ) were grown until maturity in the Cd contaminated experimental field (field C). Values shown are the means ± SD (n =3), and straws of three plants were mixed in one replication. Two asterisks indicate significant difference in comparison to WT at $P < 0.01$ by Student's *t* test.

Supplementary Table 5. Grain quality traits of *osnramp5* mutants.

Line	Amylose content (%)	Gel consistency (%)	Protein content (%)	Brown rice percentage (%)	Milled rice percentage (%)	Head rice percentage (%)
**WT**	13.0 ±0.3	94.7 ±2.5	10.2 ±0.2	76.0 ±0.6	68.5 ±0.8	55.2 ±1.8
**HZ-6-4-6**	13.1 ±0.2	94.7 ±3.1	10.7 ±0.2	76.6 ±1.3	68.2 ±2.4	57.4 ±1.4
**HZ-7-3-2**	13.1 ±0.5	96.0 ±2.0	10.7 ±0.1	73.8 ±2.4	64.6 ±1.5	53.5 ±1.3

Two mutant lines (HZ-6-4-6, HZ-7-3-2) and WT plants (HZ) were grown in the Cd contaminated experimental field (Field A). Values shown are the means ± SD (n =3). There is no significant difference in grain quality between the WT and mutant plants by Student's *t* test.

Supplementary Table 6. Major agronomic traits of *osnramp5* mutants.

Line	Plant height (cm)	Effective panicles per plant	Grains number per panicle	Seed setting rate (%)	1000-grain weight (g)	Grain yield (g/plant)	Straw weight (g/plant)
**WT**	95.41 ±1.08	14.40 ±0.67	105.66 ±4.81	69.90 ±5.16	23.83 ±0.26	22.81 ±2.38	20.52 ±1.87
**HZ-6-4-6**	94.63 ±1.42	14.66 ±0.53	103.27 ±2.64	68.08 ±5.33	23.81 ±0.40	22.06 ±1.64	19.77 ±1.52
**HZ-7-3-2**	95.06 ±4.51	13.64 ±0.93	107.30 ±4.96	65.57 ±4.11	23.54 ±0.51	21.54 ±3.31	20.86 ±2.54

Two mutant lines (HZ-6-4-6, HZ-7-3-2) and WT plants (HZ) were grown in the Cd contaminated experimental field (Field A). The survey was based on a randomized complete block design with three plot replications. Six representative plants with nearly average panicle number per plant in each plot were sampled in one replication. Values shown are the means ± SD (n =3). There is no significant difference in major agronomic traits between the WT and mutant plants by Student's *t* test.

Supplementary Table 7. Mn concentration in shoots and roots of mutant plants grown in hydroponic culture with low Mn supply.

Plant part	Line	Mn concentration (mg/kg DW)	
		2 μM Mn	4 μM Mn
Shoot	WT	267.14 ±11.95	361.78 ±23.29
	HZ-6-4-6	12.67 ±1.16**	22.60 ±2.54**
	HZ-7-3-2	13.59 ±2.43**	20.80 ±3.95**
	HZ-7-3-12	12.48 ±1.85**	22.58 ±1.64**
Root	WT	89.77 ±3.32	131.15 ±1.15
	HZ-6-4-6	21.90 ±2.56**	66.31 ±5.52**
	HZ-7-3-2	22.19 ±3.65**	63.67 ±7.48**
	HZ-7-3-12	21.67 ±3.48**	64.30 ±7.34**

Three mutant lines (HZ-6-4-6, HZ-7-3-2, HZ-7-3-12) and WT plants (HZ) were grown in normal nutrient solution for 12 d, and then transferred to the nutrient solution with 2 μM or 4 μM low Mn supply for 18 d. DW, dry weight. Values shown are the means ± SD (n =3), and three plants were mixed in one replication. Two asterisks indicate statistically significant difference in comparison to WT at $P < 0.01$ by Student's t test.

Supplementary Table 8. Primers used for sequencing and transgenic analysis.

Primer name	Primer sequence (5'-3')	Purpose
OsNramp5-F	CGGCATCAGTCAGAGGAATC	DNA sequencing
OsNramp5-R	AGGACGGAGAAATCGTGTAGAC	DNA sequencing
PS1-OS1-F	GAAGAAGGAAAAGGGACTAGC	DNA sequencing
PS1-OS1-R	TGTGAACACCACCCAAAAGCAT	DNA sequencing
PS1-OS2-F	TACACGGCACACGTCTACACAC	DNA sequencing
PS1-OS2-R	AGCAGCAAGCAATTAAGCTCATC	DNA sequencing
PS2-OS3-F	CCGATACCCACGACCACGATA	DNA sequencing
PS2-OS3-R	ACGAAGCCCCGTCTACATAAAC	DNA sequencing
PS2-OS4-F	ACTTTCGCTGGTTTTTTGGTGG	DNA sequencing
PS2-OS4-R	CCTGTGTGTTCGTTCGGAGAG	DNA sequencing
HPT-F	GCTCCATACAAGCCAACCACG	Transgenic analysis
HPT-R	CCTGCCTGAAACCGAACTGC	Transgenic analysis
CAS9-F	CGAGACGAACGGTGAGACTGGTG	Transgenic analysis
CAS9-R	GGTGCTTGTTGTAGGCGGAGAGG	Transgenic analysis
